# Inflammation and histone modification in chronic pain

**DOI:** 10.3389/fimmu.2022.1087648

**Published:** 2023-01-13

**Authors:** Wei Jiang, Li-Xi Zhang, Xuan-Yu Tan, Peng Yu, Ming Dong

**Affiliations:** ^1^ Department of Neurology and Neuroscience Center, The First Hospital of Jilin University, Changchun, China; ^2^ Department of Thyroid Surgery, The First Hospital of Jilin University, Changchun, China; ^3^ Department of Neurosurgery, The First Hospital of Jilin University, Changchun, China; ^4^ Department of Ophthalmology, The Second Hospital of Jilin University, Changchun, China

**Keywords:** chronic pain, inflammation, histone modification, gene expression, epigenetic, treatment

## Abstract

Increasing evidence suggests that epigenetic mechanisms have great potential in the field of pain. The changes and roles of epigenetics of the spinal cord and dorsal root ganglia in the chronic pain process may provide broad insights for future pain management. Pro-inflammatory cytokines and chemokines released by microglia and astrocytes, as well as blood-derived macrophages, play critical roles in inducing and maintaining chronic pain, while histone modifications may play an important role in inflammatory metabolism. This review provides an overview of neuroinflammation and chronic pain, and we systematically discuss the regulation of neuroinflammation and histone modifications in the context of chronic pain. Specifically, we analyzed the role of epigenetics in alleviating or exacerbating chronic pain by modulating microglia, astrocytes, and the proinflammatory mediators they release. This review aimed to contribute to the discovery of new therapeutic targets for chronic pain.

## 1 Introduction

Chronic pain includes inflammatory pain and neuropathic pain (NP). Inflammatory pain refers to peripheral tissue damage and persistent inflammation and neuropathic pain is a pathological adaptation of the peripheral nervous system (PNS) or central nervous system (CNS) (1). They are characterized by persistent nociceptive hypersensitivity (2). Chronic pain symptoms include an excessive increase in pain caused by painful stimuli (hyperalgesia) and pain caused by stimuli that do not usually cause pain (allodynia) (3). Current analgesics, such as opioids and nonsteroidal anti-inflammatory drugs (NSAIDS), relieve pain symptoms by inhibiting neuronal activity. However, their effects are unsatisfactory, and these drugs have serious toxicity and dose-limiting side effects (4–6). Due to the complexity of the mechanisms associated with chronic pain, researchers’ incomplete understanding of the mechanism reduces the therapeutic effect. Previous studies on the pathological mechanisms of chronic pain mainly focused on the expression of neuronal channels, receptors, and neurotransmitters (7). More research is needed on the activation of non-neurons and the expression of pro-inflammatory neuromodulators.

Neuroinflammation can occur both in the PNS and CNS. Activation of glial cells and the release and interaction of inflammatory mediators are emerging as key mechanisms for chronic pain. Local inflammatory response, including the infiltration of hematogenous immune cells and induction of various cytokines, can participate in the pathological response of chronic peripheral nerve injury. Pain can be alleviated by inhibiting the activation of inflammatory cells and the synthesis or accumulation of inflammatory factors, or by blocking the interaction between inflammatory factors and pain receptors in nociceptors. Inflammation and nerve damage can lead to the hyperexcitability of neurons. Then, a positive feedback loop between peripheral and central inflammatory cells, chemokines, and cytokines maintains neuroinflammation and chronic pain.

Accumulating evidence has shown that molecular changes in chronic pain are controlled by epigenetic mechanisms. Epigenetic modifications induce or suppresses gene expression without altering the DNA sequence, including DNA methylation, post-translational histone modifications, and expression of microRNAs (miRNAs). Histone tails are modified by acetylation and methylation. They regulate chromatin structure and function and gene expression through post-transcriptional modifications (PTMs) of the N-terminal histone tails of nucleosomes (8, 9). The histone acetyltransferase (HAT) family acts on lysine residues, leading to transcriptional activation. Conversely, the histone deacetylase family (HDACs) deacetylates histones, leading to transcriptional repression (10). Different from histone acetylation, histone methylation can lead to both gene repression and activation of gene transcription because it can act on different sites of amino acid residues. For example, methylation of Lys9 or Lys27 that occurs in histone H3 typically represses gene expression, whereas methylation of H3 Lys4, Lys36, or Lys79 typically inhibits gene activation (11). Chromatin modification is not limited to simple DNA methylation or histone modification; the activation or suppression of a gene usually involves a large number of histone co-modifications and DNA methylation.

Many studies have demonstrated that histone modifications can affect the generation and maintenance of chronic pain (12–15). The link between histone modification-induced inflammatory cells and cytokine production inhibition and pain has been reported in various disease models (16, 17). How does the inheritance of histone modifications alleviate pain by suppressing inflammation? In this review, we describe the changes in inflammatory cells and cytokines observed after inflammatory and neurological injury, which are modulated by histone modifications. Despite the increasing importance of epigenetics in the field of pain, discoveries in this area are still limited, and more research is needed to elucidate the molecular pathways behind the pain. We believe that histone modification during inflammation may provide a new therapeutic approach for treatment-resistant chronic pain.

## 2 Microglia and astrocytes: “Relay station” for epigenetic treatment of chronic pain

The role of neuroinflammation in chronic pain state is widely recognized. Microglia and astrocytes in the CNS, and satellite glia in the dorsal root and trigeminal ganglia are involved in chronic pain (18). Microglia and astrocytes, particularly microglia, are discussed in this review. Activation of microglia and astrocytes in the spinal cord and dorsal root ganglion (DRG)leads to the production of proinflammatory cytokines/chemokines, resulting in an inflammatory cascade (19). Activated microglia have both pro-inflammatory (M1) and anti-inflammatory (M2) functions. M1 microglia express pro-inflammatory molecules, whereas M2 microglia expressed anti-inflammation molecules (20). Neuroinflammation is a defense response, and anti-inflammatory cytokines and proinflammatory factors regulate these pain states. Epigenetic modifications of pro-inflammatory and anti-inflammatory factors have increased our understanding of the mechanisms underlying pain. Microglia and astrocytes have close connections to neurons and communicate with them in a bidirectional manner. Upon activation, microglia and astrocytes regulate the interaction between the nervous system and the immune system by secreting soluble mediators, including chemokines (21). This inflammatory response eventually lowers the triggering thresholds of A-σ- and C-fiber nociceptors, leading to chronic pain (22). Numerous studies have shown that microglia and astrocytes play key roles in the development of neuropathic and acute inflammatory pain (23–26). As promoters of chronic pain, non-neuronal immune cells may provide therapeutic ideas for future treatment (27).

Inflammatory mediators are involved in the production and maintenance of many forms of pain, and their importance is well-established. Chemokines are expressed in astrocytes and microglia in the spinal cord in the NP model (28). Moreover, studies have shown that the CX3CL1/CX3CR1 signaling pathway can regulate neuron-microglia interactions in the spinal cord, thus mediating the development of neuropathic pain (28, 29). Histone acetylation, deacetylation, and methylation have been shown to modulate chronic pain through inflammatory mediators (14, 30–32). Histone acetylation regulates chemokine expression in the CNS and peripheral tissues (29, 33, 34). Histone modifications, including phosphorylation, acetylation, and methylation, can occur in specific regions of the promoters of the proinflammatory cytokines tumor necrosis factor-α (TNF-α) and IL-1β and the anti-inflammatory cytokine IL-10 (35–37). Histone modifications reduce inflammation by decreasing the expression of some pro-inflammatory mediators, including IL-1β and TNF-α, and increasing the production of anti-inflammatory factors, including IL-10 (38, 39). IL-10 overexpression can reduce the expression of pro-inflammatory factor TNF-α in microglia, thereby alleviating inflammatory pain (40). On the contrary, IL-1β, IL-6, and TNF-α are involved in the activation of microglia and astrocytes (41, 42), leading to peripheral nerve injury or chronic pain caused by inflammatory responses (43–45). HDAC inhibitors have been shown to improve symptoms in several animal models of inflammatory diseases (46, 47). Data show that HDAC can be continuously expressed in mouse microglia and astrocytes, and its inhibition inhibits the neuroinflammatory response (48). Monocyte chemotactic protein-3 (MCP-3), also known as CCL7, increases after sciatic nerve ligation. MCP-3 is regulated by IL-6. Intrathecal administration of IL-6 decreased Lys27 H3 trimethylation at the McP-3 gene promoter, resulting in increased MCP-3 expression (1). Furthermore, MCP-3 expression is increased in astrocytes but not in microglia or neurons. Hence, we discuss the histone modification regulation of cytokines or chemokines through glial cells that regulate the production and maintenance of chronic pain. In recent years, numerous studies have focused on this issue. Epigenetic regulation of the activation of inflammatory cells and the expression of inflammatory mediators may be a future target for the prevention and treatment of pain.

## 3 Histone acetylation and methylation regulate chronic pain through neuro-inflammation in spinal systems

Histone acetylation primarily relies on two key enzymes, HATs and HDACs. A dynamic balance exists between HATs and HDACs to maintain stable gene expression. Nerve injury or neuroinflammatory infiltration can lead to the upregulation of histone acetylase or histone deacetylase, leading to pain. Here, we discuss the dual role and underlying mechanism of histone acetylation in the development of chronic pain. HATs reduce the electrostatic interactions between histones and DNA, thus making chromosome transcription easier. The role of HDACs is to suppress gene transcription. The molecular mechanism, in summary, involves the removal of acetyl groups, which increases the positive charge of histone tails, thereby increasing the affinity of DNA binding, leading to chromosome densification and inhibiting transcription (49, 50). Histone acetylation and deacetylation are processes of dynamic equilibrium, and when these enzymes are activated or inhibited, they lead to many diseases, including neurological dysfunction. We briefly discuss the role and possible mechanisms of histone acetylation, histone deacetylation, and histone methylation in chronic pain through neuroinflammation.

### 3.1 Histone acetylation promotes pain

Histone hypoacetylation decreases the response to nerve injury. Correspondingly, histone hyperacetylation increases the response to nerve injury, and blocking histone acetylation is effective in relieving pain (34, 49, 51). A total of 11 HDAC proteins are divided into three categories: Category I includes HDACs 1, 2, 3, and 8; Category IIa includes HDACs 4, 5, 7, and 9; Category IIb includes HDACs 6 and 10; Category IV includes HDAC 11 (52). CX3CL1 is involved in paclitaxel-induced neuropathic pain (PINP), and blocking CX3CL1 significantly inhibits microglial activation and mechanical allodynia. Inhibition of the nuclear factor-κB (NF-κB) pathway significantly inhibits H4 acetylation in the CX3CL1 promoter region and inhibits CX3CL1 upregulation, thereby alleviating pain. NF-κB can also be co-expressed with specific microglial markers; therefore, microglia may play an important role in neuropathy of pain (53). Zhang et al. demonstrated that inflammatory pain and NP resulted in increased histone acetylation levels in the brainstem nucleus raphe magnus (NRM) in a time-dependent manner (54). JNJ-26481585, a new pan-HDAC inhibitor, induces mechanical hypersensitivity in mice. In addition, IL-1 β expression in mouse macrophage RAW 264.7 was increased after JNJ-26481585 treatment *in vitro* (55). Histone acetylation can not only upregulate the pain state through inflammatory factors but also through signaling pathways in glial cells. In one study, paclitaxel-induced neuropathic pain (PINP) increased the activity of the κB subunit P65 and its binding to the IRF8 promoter region. These effects lead to the hyperacetylation of histone H3 in the IRF8 promoter region, which promotes paclitaxel-induced allodynia pain and IRF8 transcription and expression in the rat dorsal horn (19). Previous studies have shown that overexpression of IRF8 can enhance cytokines in microglia, and knockdown of IRF8 can attenuate mechanical allodynia caused by nerve injury (56).

Microglia are macrophage-like cells that reside in the central nervous system (57). Histone modification modulates pain through inflammatory intervention by microglia, similarly, acetylation regulation interferes with pain states by affecting the expression of several molecules in the chemokine system of peripheral tissue, and these chemokines can act on macrophages in peripheral tissues to affect pain (34). The C-X-C chemokine ligand type 2 [macrophage inflammatory protein 2 (MIP-2)]/C-X-C chemokine receptor type 2 (CXCR2) axis targets neutrophils and macrophages in the injured sciatic nerve (SCN) and plays an important role in neuropathic pain. High expression of these proteins is controlled by the hyperacetylation of histone H3 in neutrophils and macrophage nuclei in the SCN. HAT inhibitors can inhibit the upregulation of MIP-2 and CXCR2, thereby preventing NP (34). CCL2 and CCL3 are upregulated in macrophage immune cells in the injured SCN through lysine 9-acetylated histone H3 (H3K9Ac) and lysine 4-trimethylated H3 [H3K4me (3)]. Similarly, histone acetyltransferase inhibitors can inhibit the upregulation of CCLs and CCRs. These chemokine cascades are amplified and can be involved in chronic inflammation, causing pain after nerve injury (58).

### 3.2 Histone deacetylation promotes pain

Microglia may be involved in neuropathy of chronic pain. Treadmill running has been suggested to activate the increase in acetylated histone H3K9 in microglia, thereby promoting the production of the anti-inflammatory factor IL-10, downregulating pro-inflammatory cytokines, and ultimately improving pain (39).

D-hydroxybutyric acid (DBHB) is an HDAC inhibitor that enhances histone acetylation after spinal cord injury (SCI) in mice. DBHB can inhibit the activation of microglia and astrocyte, reduce the expression of proinflammatory factors, improve inflammatory response, and play a protective role after SCI injury (59). Similarly, HDAC inhibitors have been demonstrated to increase the acetylation of the GAD65 promoter, and global histone hyperacetylation counterbalances the inflammatory pain effect. GAD65 is used for GABA synthesis in synaptic vesicles, The downregulation of GAD65 activity leads to impaired synaptic inhibition of GABA and increased neuronal excitability, thereby promoting the occurrence of pain (60). Chronic constriction injury (CCI) can cause mechanical allodynia, hyperalgesia, and increased TNF-α levels. Oral administration of sodium butyrate, an HDAC inhibitor, significantly attenuates the pro-inflammatory cytokine TNF-α and reduces injury-related pain behaviors (61). The regulation of inflammatory factors through histone modifications is a future direction of research. Activation of JNK-c-Jun signaling was also accompanied by an increase in HDAC1 expression. A novel selective HDACI, LG325, blocked the stimulation of c-Jun phosphorylation. Double immunostaining showed that p-JNK co-localized with p-C-Jun and HDAC1 in astrocytes in the spinal dorsal horn (62). The JNK signaling pathway is important for the induction and maintenance of chronic pain (63). Similarly, HDAC2, another subtype of HDAC, is expressed in astrocytes and elevated in neuropathic pain (64), and HDAC2 mRNA is found in cortical microglia (65). In contrast, HDAC2 was shown to be unexpressed in both microglia and astrocytes in the adult mouse brain without neuropathic pain (66). These results suggest that HDAC may inhibit the expression of pain related genes in astrocytes. Cell-specific expression in the CNS may be responsible for this phenomenon. Attenuating pain by reducing the production of pro-inflammatory factors may be a good target in the future. Whether HDAC can be used as a target for the treatment of chronic pain through glial cells still needs to be studied.

HATs and HDACs seem to have different effects on chronic pain. There are many factors responsible for this difference, such as different doses and cell types, which lead to different effects of HDAC inhibitors. TSA, as a broad-spectrum HDAC inhibitor, has different pro-inflammatory and anti-inflammatory effects on macrophages within a certain concentration range (67). Furthermore, with respect to intrathecal injection, oral administration is not as effective as local injection, and there are more unknown influencing factors (68–70). Histone modification is a complex process. Most importantly, histone acetylases or deacetylases do not increase or decrease NP alone but act as acetyl agents to affect chronic pain by increasing or decreasing the expression of specific genes. Acetylation can bind to promoters of different genes, leading to acetylation or deacetylation of genes, and the product of gene expression determines whether chronic pain is aggravated or alleviated. Therefore, understanding the acetylation or deacetylation of corresponding gene promoters caused by histone acetylases or deacetylases can provide new guidance for the treatment of chronic pain. We should focus on specific HDACs and HATs in the future, hoping to treat pain by targeting different cells or even different genetic targets using highly specific inhibitors.

Although histone acetylation and deacetylation are effective in alleviating chronic pain symptoms, they have major limitations. First, most inhibitors act both centrally and peripherally, raising concerns regarding drug toxicity. Second, many of these compounds require long-term, high-dose treatment that increases side effects and reduces patient compliance. These factors should be considered when using these compounds in the context of pain. Histone acetylation is not only involved in HATs and HDACs but may also involve other epigenetic forms. Its complex and highly cell-specific nature suggests that we still have a long way to go (71). Therefore, we cannot simply assume that histone acetylation and deacetylation affect gene transcription alone. This is also what we discussed above: HDAC can have two interactive ways on chronic pain.

### 3.3 Histone methylation regulates pain

Histone methyltransferases and demethylases are also altered in inflammation-mediated neuropathic pain. G9a, a histone H3K9 dimethyl transferase, catalyzes H3K9 dimethylation (H3K9me2), which is associated with gene silencing (72). Inhibition of G9a can reverse Mu opioid receptor (MOR, encoded by *Oprm1*) downregulation in the DRG induced by spinal nerve ligation (SNL) and enhances the effect of morphine on pain hypersensitivity induced by nerve injury (73). Enhancer of zeste homolog 2 (EZH2) leads to gene silencing by catalyzing di-methylation and tri-methylation of histone H3 on lysine 27 (H3K27Me2/3) and has been demonstrated to be expressed in microglia (74). As an EZH2 inhibitor, EPZ-6438 controls important inflammatory gene targets by regulating the promoter levels of interferon regulators and transcriptional activators. Therefore, EPZ-6438 may be an effective treatment for neuroinflammatory diseases associated with microglial activation (75). After partial sciatic nerve ligation (PSL), the number of microglia expressing EZH2 increased significantly in the spinal cord of rats (37). Activation of microglia leads to increased overall levels of EZH2 and tri-methylated H3K27 in the L4 and L5 spinal dorsal horn, and excessive production of TNF-α and IL-β in the spinal dorsal horn contributes to NP. Intrathecal injection of an EZH2 inhibitor can relieve chronic pain (37). MCP-3 (CCL7) expression increased in IL-6 sensitive astrocytes after CCI in mice. This was due to the reduced H3K27 trimethylation of the MCP-3 promoter. Increased MCP-3 expression activates spinal microglia expressing CCR2, thereby inducing central sensitization. The CCR2 receptor expressed in spinal cord neurons and microglia may be induced by MCP3, thereby enhancing neuronal sensitivity (76).

Granulocyte macrophage colony-stimulating factor (GM-CSF) is a key pro-inflammatory cytokine. In mouse models of arthritis and inflammatory pain, GM-CSF up-regulated IRF4 expression by enhancing JMJD3 (histone H3K27 demethylase) activity (77, 78). IRF4 regulates the formation of CCL17 and then mediates the proinflammatory and analgesic effects of GM-CSF. After SCI, JMJD3 expression is upregulated and MMP-3 and MMP-9 gene expressions are regulated. In addition, inhibition of JMJD3 inhibited the expression of matrix metalloprotease-3 (MMP-3) and MMP-9 genes and significantly reduced the permeability of the blood-spinal cord barrier (BSCB) (79). Subsequently, increased JMJD3 expression in the cauda equina of rats with lumbar spinal stenosis-induced chronic NP was demonstrated. JMJD3 infiltrates cauda equina macrophages after injury, mediates neuroinflammation, and increases the permeability of the blood-nerve barrier (80). We summarize the key studies focusing on the links between histone modification with chronic pain in **Table 1**. These results suggest a potential therapeutic approach for the treatment of inflammatory pain and NP.

**Table 1:** Roles of histone modification in chronic in rodent models.

Pain model	Positive	Type of histone modification	Alteration after injury	Molecular effect (Positive control ↑ and negative control ↓)	Inhibition (-) or activation (+) to histone modification	Nociceptive behavior response to inhibitors	Ref.
PINP	Microglial	Acetylation of H4	Increased	CX3CL1 (↑)	NF-κB inhibitor (-)	Relieve	(53)
JNJ-26481585 (HDAC inhibitors) induced	Macrophage	Acetylation	Increased	NF-κB and IL-β(↑)	gabapentin (-)	Relieve	(55)
PINP	Microglial	Acetylation of H3	Increased	NF-κB (↑)	MDA7 (-)	Relieve	(19)
NP	Neutrophils and macrophage	Acetylation of H3	Increased	CXCR2(↑)	HAT inhibitor (-)	Relieve	(34)
NP	Macrophage	Acetylation of H3 and trimethylation of H3	Increased	CCL2 and CCL3(↑)	HAT inhibitor (-)	Relieve	(58)
NP	Microglial	Acetylation of H3	Decreased	/	treadmill running (+)	Relieve	(39)
Spinal cord injury	Microglial and glial	Acetylation of H3	Decreased	NLRP3 inflammasome (↑)	DBHB (+)	Relieve	(59)
NP	Sciatic nerve	Acetylation	Decreased	TNF-α (↑)	HDAC inhibitor (+)	Relieve	(61)
NP	Astrocytes	Acetylation	Decreased	c-Jun (↑)	HDAC inhibitor (+)	Relieve	(62)
NP	DRG	Dimethylation of H3	Increased	MOR (↑)	Histone methyltransferase inhibitor (-)	Relieve	(73)
NP	Microglia	Trimethylation of H3	Increased	TNF-α, IL-1β, and MCP-1(↑)	Histone methyltransferase inhibitor (-)	Relieve	(37)
NP	Microglia and astrocytes	Trimethylation of H3	Decreased	IL-6 (↓)	MCP-3 antibody (-)	Relieve	(76)

↑ means positive control to molecular, ↓ means negative control to molecular. (+) means activation to histone modification, (-) means Inhibition to histone modification.

## 4 Conclusions and future studies

It is clear that we are at this initial stage, where there is no consensus in the literature and some contradictory findings have been described. In the field of pain, the contributions of genetic and epigenetic mechanisms have been widely recognized. We believe that the epigenetic mechanism of chronic pain will be further explored in the future and there is great potential for the epigenetic intervention of inflammatory system genes to improve pain. Currently, our discussion of rodent models, which poses a challenge for clinical transformation, is still in the preclinical stage. Further studies on epigenetics and neuroinflammation are needed to find preclinical transformational management of neuropathic pain. Epigenetic mechanisms controlling chronic pain may offer a wide range of potential therapeutic targets. As a potential targeted intervention, epigenetic inheritance may improve the symptoms of chronic pain by suppressing inflammation, preventing injury sensitization.

## Author contributions

WJ and X-YT designed the study, reviewed the literature, and drafted the manuscript. L-XZ helped in writing and revising the manuscript. PY and MD contributed significantly to the revision of the final manuscript. All authors contributed to the article and approved the submitted version.
